# Supplementary material for: The effects of department of Veterans Affairs medical centers on socio-economic outcomes: Evidence from the Paycheck Protection Program
Source: PLoS One. 2022 Dec 22;17(12):e0269588. doi: 10.1371/journal.pone.0269588 (PMC9778558; doi:10.1371/journal.pone.0269588)
Supplement: S1 Appendix — (PDF) [file pone.0269588.s001.pdf]

Online Appendix

Data Description

- Zipcode Demographics
  - Age Related Features
    * Share of individuals under the age of 18.
    * Share of individuals between age 18 to 24.
    * Share of individuals between age 25 to 44.
    * Share of individuals between ages 45 and 64.
    * Share of individuals over the age of 65.
  - Education Related Features
    * Share of individuals with less than a high school degree.
    * Share of individuals with some college.
    * Share of individuals with a college degree.
    * Share of individuals with over a college degree.
  - General Healthcare Features
    * Share of individuals with private health insurance.
    * Share of individuals with public health insurance.
  - Industry Related Features
    * Share of individuals in finance, insurance, and real estate.
    * Share of individuals in agriculture, mining, forestry, and fishing.
    * Share of individuals in arts and entertainment.
    * Share of individuals in construction.
    * Share of individuals in education and health.
    * Share of individuals in information services.
    * Share of individuals in manufacturing.
    * Share of individuals in other services.
    * Share of individuals in professional services.
    * Share of individuals in public administration.
    * Share of individuals in retail trade.
    * Share of individuals in utilities and transportation.
    * Share of individuals in wholesale trade.
  - Occupation Related Features
    * Share of individuals in building and grounds cleaning and maintenance.
    * Share of individuals in construction, extraction, and Maintenance.
    * Share of individuals in farming, fishing, and forestry.
    * Share of individuals in food preparation and serving related.
    * Share of individuals in healthcare support.
    * Share of individuals in management, business, and financial operations.
    * Share of individuals in office and administrative support.
    * Share of individuals in personal care and service.
    * Share of individuals in professional and related.
    * Share of individuals in production.
    * Share of individuals in protective services.

    - Share of individuals in sales and related.
    - Share of individuals in transportation and material moving.
  - Population Related Features
    - Total population
  - Race Related Features
    - Share of Blacks.
    - Share of Whites.
  - Poverty Related Features
    - Share of individuals under the poverty line between ages 18 and 64.
    - Share of individuals under the poverty line over the age of 65.
    - Share of individuals under the poverty line under the age of 18.
    - Share of individuals under the poverty line who are White.
  - Sex Related Features
    - Share of males. male
    - Overall unemployment rate.
    - Unemployment rate for females.
    - Unemployment rate for males.
  - Veteran Share Related Features
    - Share of individuals who are Veterans between ages 18 and 64.
    - Share of individuals who are Veterans over the age of 65.

- VA Facilities
  - VA Facility Features
    - Average wait for a primary care appointment for a regular patient at a Large Regional VA Facility
    - Average wait for a primary care appointment for a new patient at a Large Regional VA Facility
    - Average wait for a specialty care appointment for a regular patient at a Large Regional VA Facility
    - Average wait for a specialty care appointment for a new patient at a Large Regional VA Facility
    - Percent who say they usually or always get a non-urgent primary care appointment when they need it at a Large Regional VA Facility
    - Percent who say they usually or always get an urgent primary care appointment when they need it at a Large Regional VA Facility
    - Percent who say they usually or always get a non-urgent specialty care appointment when they need it at a Large Regional VA Facility
    - Percent who say they usually or always get an urgent specialty care appointment when they need it at a Large Regional VA Facility
    - Average wait for a primary care appointment for a regular patient at a Local VA Facility
    - Percent who say they usually or always get a non-urgent primary care appointment when they need it at a Local VA Facility
    - Interaction: Percent who say they usually or always get a non-urgent primary care appointment when they need it at a Local VA Facility and Distance to closest Local VA Facility

    * Interaction: Average wait for a primary care appointment for a regular patient at a Local VA Facility and Distance to closest Local VA Facility
    * Interaction: Percent who say they usually or always get a non-urgent primary care appointment when they need it at a Local VA Facility and Average wait for a primary care appointment for a regular patient at a Local VA Facility
    * Average travel time to Local VA Facility
    * Distance to closest Large Regional VA Facility
    * Distance to closest Local VA Facility

- Zipcode and VA Facility Interactions
  - Zipcode and VA Facility Interaction Features
    * Each VA Facility Feature interacted with each Zipcode Demographics Feature

- We use the Joint Economic Committee (JEC) measure of social capital at the county-level [30], which contains indicators "related to family structure and stability, family interaction and investment, civil society, trust and confidence in institutions, community cohesion, institutions, volunteerism, and social organization." These indicators, at the county-level, include: the share of births in the past year to women who were unmarried, the share of women ages 35-44 who are currently married and not separated, the share of own children living in a single-parent family, registered non-religious non-profits per 1,000, religious congregations per 1,000, an informal civil society sub-index, and the average of votes in the Presidential election per citizen ages 18 and over. These data are generally compiled based off of estimates from 2012 to 2016—well before COVID-19 and, therefore, predetermined with respect to current infection rates and the spread of the virus.

Evaluating Potential Measurement Error

To assess the representativeness of our data on Veterans contained in the PPP data, we also draw on detailed demographic data from the 2014 to 2018 American Community Survey (ACS) implemented through the Census Bureau, including (but not limited to): the age, education, race, industry, and occupation distributions, median household income, median housing values, population, unemployment rate, gender, marriage rates. We obtain these data at the zipcode-level.

Table A.1 regresses important proxies for the productivity of a location—logged median household income, logged median housing values, and the unemployment rate—on an indicator for whether the zipcode has an above the median share of missing Veteran responses. That is, we ask whether the incidence of missing Veteran responses is correlated with characteristics that proxy for the types of unobserved characteristics that could confound our subsequent predictive models.

We consider two sets of specifications for each outcome variable: with and without a weight. We weight by the number of PPP recipients in a zipcode since it is the most transparent and intuitive way of reducing noise. For example, if only 10 people in a zipcode received a PPP loan, it is likely that there will be no Veteran recipients, let alone that a recipient may actually state that they are a Veteran (versus leaving it missing in the form).

When we fail to weight by number of responses, we see that zipcodes with higher rates of missing Veteran responses have 1% higher income, 3.1% higher housing values, and 0.2 percentage points higher unemployment rates. However, after introducing our

weight, we find no statistically significant differences for household income, 2.2% lower housing values, and a 0.1 percentage point lower unemployment rate. In this sense, while Veteran responses appear to be missing in ways that are correlated with location-specific characteristics, these differences are economically minor and tend to disappear after using our weights.

Table A.1. Examining Whether Veteran Status is Missing at Random

**Dep. var. =**	**log(Household Income)**		**log(Housing Value)**		**Unemployment Rate**	
High Missing Veterans %	.010***	.002	.031***	-.022**	.002***	-.001**
	[.003]	[.004]	[.005]	[.009]	[.001]	[.000]
R-squared	.65	.82	.63	.67	.17	.44
Sample Size	29856	29856	29642	29642	30989	30989
Controls	Yes	Yes	Yes	Yes	Yes	Yes
Has Weight	No	No	No	No	No	No

Notes.—Sources: Small Business Administration Paycheck Protection Program (PPP) and 2014-2018 American Community Survey. The table reports the coefficients associated with regressions of logged median household income, logged median housing values, and the unemployment rate at a zipcode-level on an indicator for whether the zipcode has a high rate of missing Veteran responses, conditional on zipcode controls. Controls include: logged population, the age distribution (the share of individuals under age 18, 18-24, 45-64, 65+), the share married, the education distribution (the share with less than a high school degree, some college, and college plus), and the industry distribution (the share in agriculture / mining / forestry, construction, manufacturing, wholesale trade, retail trade, transportation, information services, finance / real estate / insurance, professional services, education / health, arts / entertainment, other services, and public administration). Standard errors are clustered at the zipcode-level and observations with the weight are using the number of respondents in the zipcode.

As an additional exercise to assess the data quality, Fig A.1 shows a binned scatterplot of the actual share of Veterans ages 18 and older in a zipcode with the share implied by the PPP data. Given the fact that many PPP recipients did not report Veteran status, combined with the fact that the coronavirus has had a differential effect on Veterans and non-Veterans, there is little reason to suspect that the two shares will be equal in levels. Their correlation is 0.30 (0.08) when we weight (unweight) zipcodes by the number of PPP recipients in them.

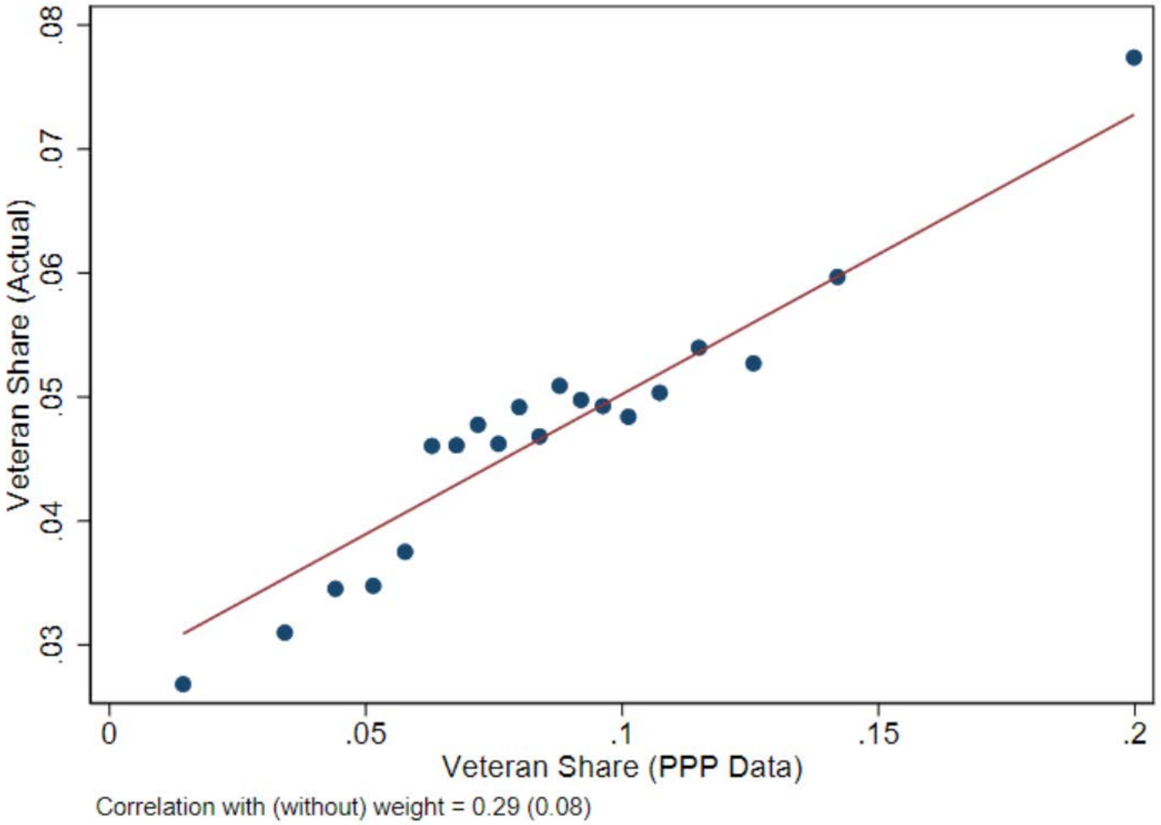

Fig A.1. Comparison of Actual and Sample Veteran Zipcode Shares
Sources: Small Business Administration Paycheck Protection Program (PPP) and 2014-2018 American Community Survey. The figure reports the share of Veterans in a zipcode from the ACS and the share of Veterans from the PPP data with and without weights where we use the number of PPP recipients in an area as the weight.
