# Supplementary material for: The effects of department of Veterans Affairs medical centers on socio-economic outcomes: Evidence from the Paycheck Protection Program
Source: PLoS One. 2022 Dec 22;17(12):e0269588. doi: 10.1371/journal.pone.0269588 (PMC9778558; doi:10.1371/journal.pone.0269588)
Supplement: Attachment — Submitted filename: Response to Reviewers.pdf [file pone.0269588.s002.pdf]

Dear editor and anonymous referees,

Thank you so much for your thoughtful review. We go point-by-point in response to the questions raised below, and we have overhauled the paper into the right PLOS One format.

Reviewer #1:

The reviewed article raises an interesting and timely issue - the impact of the global Covid-19 pandemic on veterans, a group particularly vulnerable to the effects of the pandemic due to their age. The work is quite carefully done, although I have a few comments:

1) the title is too general and does not reflect the scope of the work. It should be made clear that the study is about veterans, as the current form promises a broader scope of content

Great point—thank you. We updated the title to: "The Effects of **Department of Veterans Affairs** Medical Centers on Socio-economic Outcomes: Evidence from the Paycheck Protection Program."

2) phrases like "While we are not the first to study the Paycheck Protection Program" sound too colloquial for an academic paper

Thank you—we went through again and polished the wording, including in that location.

Reviewer #2:

---

1. The literature review lacks studies investigating the relations between healthcare services and economic performance. While this relation is rather not straightforward and not intuitive

I encourage authors to enrich this section.

That's a great point. We now incorporate a few papers about how healthcare services leads to the promotion of social capital, which feeds directly into economic performance, and how a healthy and physically well workforce is more capable and agile. That depends in part on access.

2. You write that about 80% of race information and 75% of status is missing in the dataset. So maybe the dataset is not representative. E.g. maybe PPP loans are more likely given to the wealthier and white veterans as they might be overrepresented in your dataset?

It's a great question and concerned us too. Fortunately, we have the universe of PPP loans. Unfortunately, many people do not put in their race and veteran status information. However, race and veteran status have no correlation (p-value = 0.97). When we restrict the sample to all the non-missing observations, we have an extremely small sample of 140 people: here, we see a strong positive correlation between being black and being a veteran, which would bias us in the other direction of seeing more veteran minorities (versus white veterans).

3. You don't sufficiently explain why PPP loans are more likely given to veterans?

Please provide a logical explanation taking into account your results.

In my opinion, you overestimate the role of VA Medical Centers in the development of social capital.

E.g. the visits at these facilities are not so regular to create social ties. Moreover, the primary aim is healthcare, not chats.

Am I wrong?

You are exactly right with the intuition for why someone goes into a VAMC. However, that's the interesting result: VAMCs are predictive of these socio-economic outcomes too. We talked internally in the Department of Veterans Affairs and we found a lot of interest and support for the fact that veterans often talk about much more than just health – they do become communal places to a largest extent, at least the "good" and high quality VAMCs. That is what the VAMC ratings help establish – a measure of the quality, which spills over into the communal aspect.

4. What is the relation between the VA Medical Centers ratings and PPP loans?

As I don't see the connection, please provide a logical explanation.

One possible explanation that we have heard some anecdotal evidence about is that VAMCs, because they do more than just healthcare (they are a place where veterans gather), provide a venue for information dissemination. Because receipt of the PPP was so heavily dependent on information and banking relationships, veterans may have received recommendations, tips, and connections through their VAMC community. While it is also possible that areas with better VAMCs simply are more likely to receive PPP overall, we show that there is unique predictive power of the VAMCs—that is, we estimate the models with and without the VAMC ratings.
